# De novo variant in RING finger protein 213 causes systemic vasculopathy

**DOI:** 10.1172/jci.insight.190094

**Published:** 2025-06-09

**Authors:** Ayako Kashimada, Tomoko Mizuno, Eriko Tanaka, Susumu Hosokawa, Tomohiro Udagawa, Yuichi Hiraoka, Keisuke Uchida, Tomohiro Morio, Kenjiro Kosaki, Masatoshi Takagi

**Affiliations:** 1 Department of Pediatrics and Developmental Biology, Institute of Science Tokyo, Tokyo, Japan.; 2 Department of Pediatrics, Kyorin University, Tokyo, Japan.; 3 Laboratory of Molecular Neuroscience and; 4 Laboratory of Genome Editing for Biomedical Research, Medical Research Institute, Institute of Science Tokyo, Tokyo, Japan.; 5 Department of Pathology, Institute of Science Tokyo Hospital, Tokyo, Japan.; 6 Center for Medical Genetics, Keio University, Tokyo, Japan.

**Keywords:** Development, Vascular biology, Genetic diseases, Innate immunity

## Abstract

Systemic arterial stenosis, including moyamoya disease (MMD) and middle aortic syndrome (MAS), is a rare condition of unclear etiology. MMD is a cerebral angiopathy, and MAS affects the abdominal and thoracic aorta. Although some genetic associations with MAS have been identified, the causes remain elusive. In this study, de novo heterozygous missense variants of RING finger protein 213 (*RNF213*) (p.His4058Pro and p.Thr4155Pro) in 2 unrelated families with MAS and MMD were studied by whole-exome sequencing. To elucidate the significance of these variants, we produced knockin mice carrying the *Rnf213* p.His4058Pro variant. Homozygous knockin mice exhibited perinatal lethality because of respiratory failure and lung dysplasia, suggesting that this variant is pathogenic. Lung dysplasia in homozygous knockin mice was associated with upregulated innate immunity and inflammatory responses and downregulated cell proliferation. These findings suggested that in mice, the *RNF213* p.His4058Pro variant plays critical roles in regulation of innate immunity and inflammation that affect lung development, revealing the complexity of *RNF213* function in various tissues and species. In conclusion, this study provides insights into the genetic basis of MAS and MMD, highlights the potential involvement of *RNF213* variants in systemic vasculopathy, and identifies unexpected associations with lung development and immune processes.

## Abstract 2

Genetic variants in RNF213 are linked to rare vascular diseases, impacting immunity, inflammation, and lung development, offering insights into systemic vasculopathy and its underlying mechanisms.

## Graphical abstract

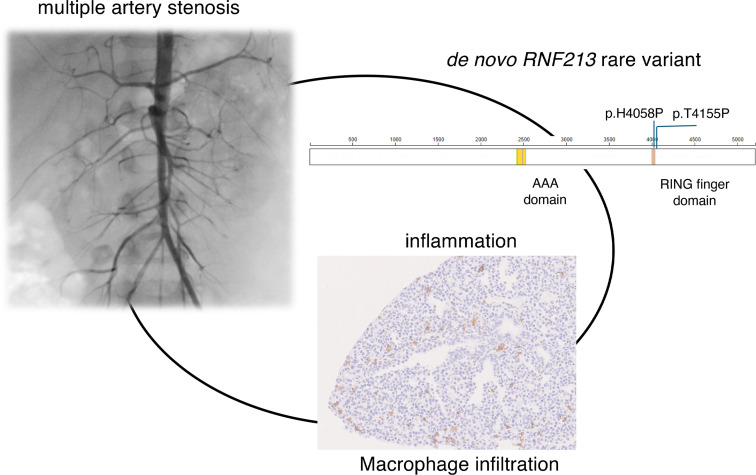

## Introduction

Moyamoya disease (MMD) is a rare progressive cerebral angiopathy characterized by bilateral internal carotid artery stenosis and abnormal collateral vessels. Immune inflammation–related responses may be an essential triggering factor of MMD (1). RING finger protein 213 (*RNF213*) was identified as a susceptibility gene in a genome-wide association study in East Asian populations (2). *RNF213* was also reported as a causative gene for pulmonary arterial hypertension and for peripheral pulmonary artery stenosis (3, 4). Middle aortic syndrome (MAS) is a rare disease that occurs in children and young adults, accounting for 0.5%–2% of all cases of aortic stenosis (5). MAS presents as segmental or diffuse narrowing of the abdominal and/or distal descending thoracic aorta, with variable involvement of the renal and visceral branches (6), leading to renovascular hypertension, hypertensive encephalopathy, stroke, and progressive cardiac and renal dysfunction in severe cases (7–9). Several cases of systemic arteriopathy combined with MMD have been reported (10, 11). Renal artery stenosis is the most common extracranial arteriopathy in pediatric MMD, with a prevalence of 5%–8% (12, 13); however, the pathogenesis of whole-body vasculopathy involving MMD and MAS is unclear. The etiology of MAS has been described in association with genetic syndromes (15%), such as neurofibromatosis (von Recklinghausen disease) type I, Alagille syndrome or Williams syndrome, and inflammatory diseases (17%), such as Takayasu’s arteritis; however, most cases are idiopathic (6). Inflammation is one of the etiologic factors associated with MAS (6); therefore, it is one of the key critical factors in the development and progression of MMD and MAS. Recently, whole-exome sequencing (WES) in 35 families with MAS revealed variants in genes associated with vascular disease, such as *NF1*, *JAG1*, *ELN*, *GATA6*, and *RNF213* (9). *NF1* is responsible for neurofibromatosis, *ELN* for Williams-Beuren syndrome, *JAG1* for Alagille syndrome, and *GATA6* for persistent truncus arteriosus. However, no functional analysis has been performed on disease-causing genes for MAS. In this study, we have identified de novo heterozygous missense *RNF213* variants in 2 unrelated families with both MAS and MMD and demonstrated the pathogenicity of the variant. This is the first molecular study to our knowledge demonstrating that a de novo variant of *RNF213* induces pro-inflammatory events and causes systemic MAS and MMD.

## Results

### Clinical information of patients with MAS and MMD.

**Figure 1:**
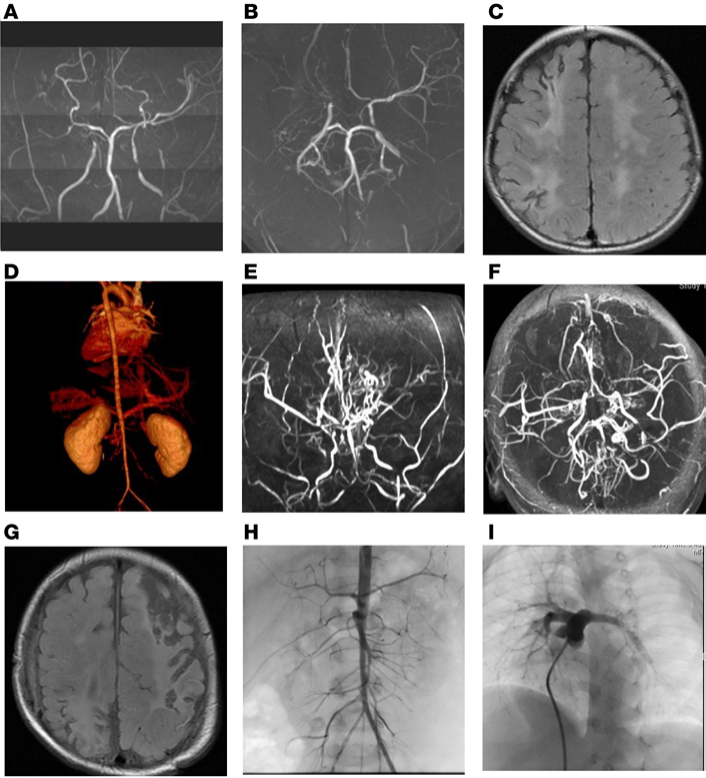
Clinical imaging data of the patients. Upper. Patient 1 at 4 years and 3 months. Magnetic resonance angiography (MRA) frontal (**A**) and axial (**B**) views of the cerebral artery show bilateral severe stenosis of the internal carotid artery. The right anterior and right middle cerebral arteries are not detected. Brain atrophy of the right cerebral hemisphere and bilateral high intensity on white matter lesion on FLAIR-MRI (**C**). Three-dimensional CT angiography at 2 years and 10 months shows narrowing across the thoracoabdominal aorta to the iliac artery (**D**). Middle. Patient 2 at 1 year and 5 months. MRA frontal (**E**) and axial (**F**) views of the cerebral artery show bilateral severe stenosis of the internal carotid artery and bilateral vertebral artery to basilar artery stenosis. Brain FLAIR-MRI at 1 year and 8 months shows old cerebral infarcts in the left frontoparietal and right temporal and occipital lobes (**G**). Angiography at 1 year and 11 months showed stenosis of the abdominal aorta and bilateral renal arteries (**H**) as well as peripheral pulmonary artery stenosis (**I**).

Patient 1 was a 9-year-old boy. He experienced unilateral or generalized convulsions at 1 month of age and cerebral infarction at 14 months of age and was diagnosed with MMD (right grade 3, left grade 2) (Figure 1, A–C). CT angiography of the patient at the age of 2 years and 8 months revealed multiple artery stenosis across the internal cervical and vertebral arteries, thoracic and abdominal aortas, renal artery, and iliac artery (Figure 1D). At the age of 5 years 5 months, encephaloduroarteriosynangiosis on the right side and encephaloduroperiostealsynangiosis on the left side were performed for revascularization.

Patient 2 was a 6-year-old boy. At 2 months of age, he had unilateral or generalized convulsions because of cerebral infarction and hemorrhage and was diagnosed with MMD. MRA of the patient at the age of 17 months revealed multiple artery stenosis across the internal carotid and vertebral arteries (Figure 1, E and F). At 20 months, severe old cerebral infarcts were detected through MRI (Figure 1G). Encephaloduroarteriosynangiosis was performed on the right side and encephaloduroperiostealsynangiosis on the left side. Angiography at 2 years 11 months showed stenosis of the abdominal aorta, bilateral renal arteries, and iliac artery and peripheral pulmonary arterial stenosis (Figure 1, H and I).

### Genetic characterization of patients with MAS and MMD.

De novo heterozygous missense *RNF213* variants were identified by trio-based WES in the patients with MAS and MMD from 2 unrelated families.

**Figure 2:**
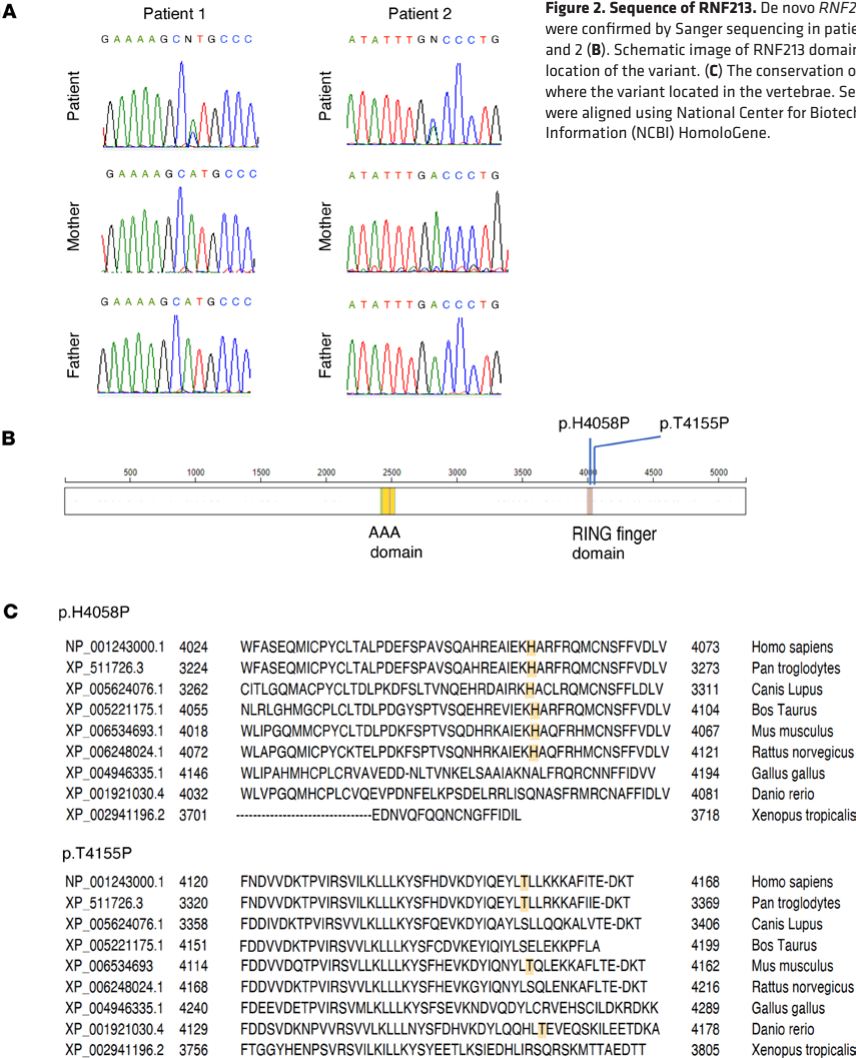
Sequence of RNF213. De novo *RNF213* variants were confirmed by Sanger sequencing in patients 1 (**A**) and 2 (**B**). Schematic image of RNF213 domains and location of the variant. (**C**) The conservation of the codon where the variant located in the vertebrae. Sequences were aligned using National Center for Biotechnology Information (NCBI) HomoloGene.

Patient 1 has a de novo heterozygous missense *RNF213* variant (NM_001256071: c.12173A>C, p.His4058Pro [H4058P]) (Figure 2A). This variant was absent from the exome aggregation consortium (ExAC) database and Genome Aggregation Database (gnomAD) v4.1.0. Combined Annotation Dependent Depletion (CADD) score was 23.3.

Patient 2 has a de novo heterozygous missense *RNF213* variant (NM_001256071: c.12463A>C, p.Thr4155Pro [T4155P]). This variant is absent from the ExAC database and gnomAD v4.1.0. CADD score was 14.4 (Figure 2A). p.H4058P was in the RING finger domain (aa 4042–4092), and p.T4155P was in the region distal to the RING domain (Figure 2B). The positions of both codons were conserved across mammals (Figure 2C).

### Phenotypic analysis of Rnf213 H4008P heterozygote knockin mice.

To define the effect of the p.H4058P variant, the *Rnf213*-knockin (*Rnf213*-KI) mouse model carrying a heterozygous histidine-to-proline substitution (H4008P), corresponding to that of the human H4058P, was generated by CRISPR/Cas9 genome editing. *Rnf213^WT/H4008P^* mice showed no abnormalities in appearance and anatomical structure of internal organs. MRA did not reveal abnormalities in the cerebral arteries, aortic arch, and descending aorta (Supplemental Figure 1, A–H; supplemental material available online with this article; https://doi.org/10.1172/jci.insight.190094DS1). The aortic ring assay using VEGF-induced microvascular sprouting from aorta embedded in collagen gel culture (14) showed no obvious differences in microvascular development (Supplemental Figure 2, A and B), indicating that *Rnf213^WT/H4008P^* does not show obvious phenotypes in mice.

### Rnf213^H4008P/H4008P^ mice have perinatal lethality due to respiratory failure.

**Figure 3:**
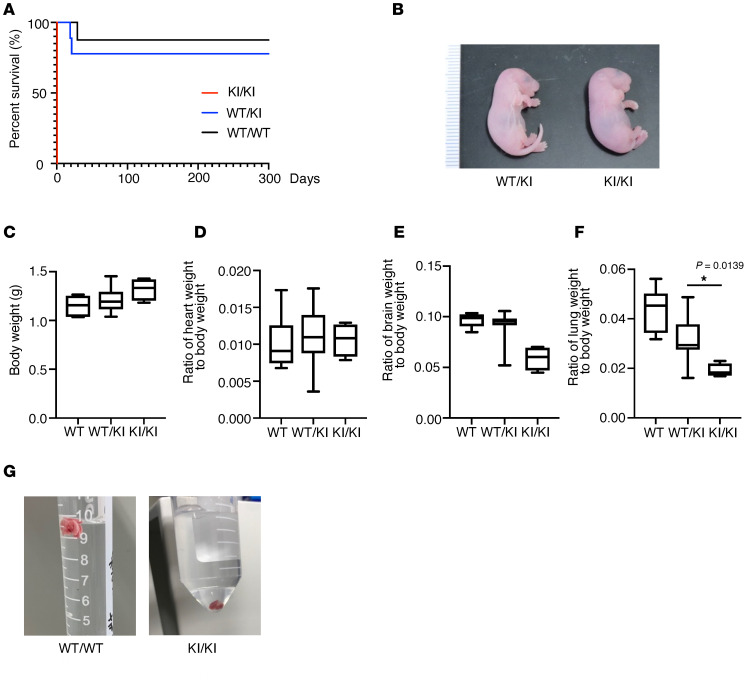
Phenotypic comparison of *Rnf213^WT/WT^* and *Rnf213^WT/H4008P^* and *Rnf213^H4008P/H4008P^* littermates on P0. (**A**) Kaplan-Meier survival curves of *Rnf213^WT/H4008P^* littermate mice. The littermates were fostered by surrogate mothers (WT/WT, *n* = 12; WT/KI, *n* = 12; and KI/KI, *n* = 4). (**B**) External appearance of P0 *Rnf213^WT/H4008P^* and *Rnf213^H4008P/H4008P^* mice. scale bar: 1 mm. (**C**) Comparison of body weight between genotypes (WT/WT, *n* = 7; WT/KI, *n* = 24; and KI/KI, *n* = 4). (**D**) Comparison of heart weight between genotypes (WT/WT, *n* = 6; WT/KI, *n* = 18; and KI/KI, *n* = 4). (**E**) Comparison of brain weight between genotypes (WT/WT, *n* = 7; WT/KI, *n* = 15; and KI/KI, *n* = 4). (**F**) Comparison of lung weight between genotypes (WT/WT, *n* = 7; WT/KI, *n* = 19; and KI/KI, *n* = 4). (**G**) Whole lungs were placed in PBS at P0. The lungs of WT/WT mice floated, and those of KI/KI mice sank at the bottom of the PBS. Data are presented as mean ± SD. Box plots show the interquartile range, median (line), and minimum and maximum (whiskers). *P < 0.05. WT/WT, *Rnf213^WT/WT^* mice; WT/KI, *Rnf213^WT/H4008P^* mice; KI/KI, *Rnf213^H4008P/H4008P^* mice.

Although *Rnf213^WT/H4008P^* mice did not mimic human disease phenotypes, all *Rnf213^H4008P/H4008P^* mice born from *Rnf213^WT/H4008P^* mice died shortly after birth, and 66% of *Rnf213^WT/WT^* and 72% of *Rnf213^WT/H4008P^* offspring born from *Rnf213^WT/H4008P^* mice died within 2 weeks of birth (Supplemental Figure 3). The number of *Rnf213^WT/WT^*, *Rnf213^WT/H4008P^*, and *Rnf213^H4008P/H4008P^* embryos was approximately consistent with the Mendelian ratio. Reduced survival of *Rnf213^WT/WT^* and *Rnf213^WT/H4008P^* offspring born to *Rnf213^WT/H4008P^* mice was because the *Rnf213^WT/H4008P^* mothers were not rearing their litters properly. Consequently, surrogate mothers were used. After swapping the *Rnf213^WT/H4008P^* mother mouse with a surrogate mother, only 1/12 *Rnf213^WT/WT^* and 2/12 *Rnf213^WT/H4008P^* mice died. However, despite the use of surrogate mothers, all *Rnf213^H4008P/H4008P^*-born mice died shortly after birth (Figure 3A). *Rnf213^H4008P/H4008P^* neonatal mice showed no external abnormalities (Figure 3B) and no significant differences in body weight, heart weight, and brain weight (Figure 3, C–E); however, their lungs were significantly smaller than those of *Rnf213^WT/WT^* and *Rnf213^WT/H4008P^* mice (Figure 3F). The lung of an *Rnf213^WT/WT^* neonate mouse was inflated and floated in phosphate-buffered saline (PBS), whereas the lung of an *Rnf213^H4008P/H4008P^* mouse sank (Figure 3G). Therefore, we hypothesize that death was due to postnatal respiratory failure.

### E18.5 Rnf213^H4008P/H4008P^ mice have disorganized lung structure.

**Figure 4:**
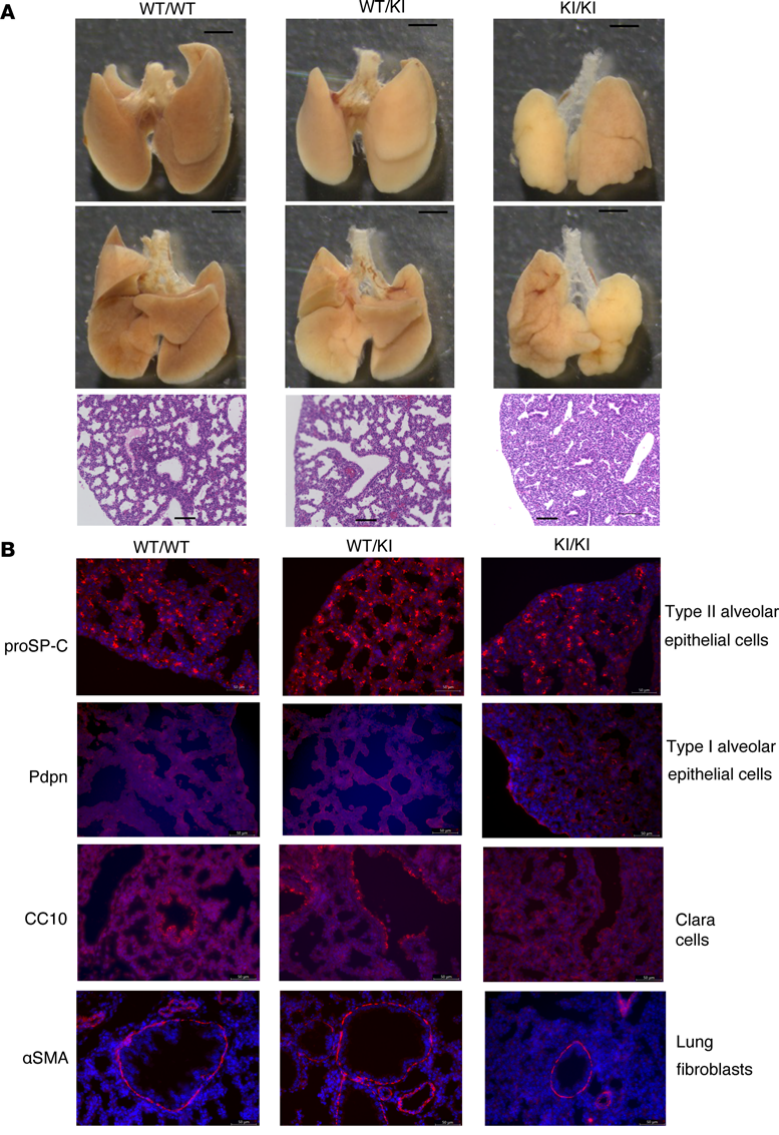
Phenotypic analysis of E18.5 *Rnf213^H4008P/H4008P^* mice reveals abnormal lung appearance. (**A**) Morphology of lung tissue and alveoli in E18.5 *Rnf213^WT/WT^* and *Rnf213^WT/H4008P^* *Rnf213^H4008P/H4008P^* mice. Whole lungs (upper: frontal view and middle: back view; scale bar: 1 mm) and H&E-stained lungs (lower: 20× original magnification, scale bar: 100 μm). (**B**) Representative images showing immunohistochemistry of pro-surfactant protein C (proSP-C), podoplanin (Pdpn), club cell secretory protein (Cc10), and α–smooth muscle actin (α-SMA) in E18.5 *Rnf213^WT/WT^* and *Rnf213^WT/H4008P^* *Rnf213^H4008P/H4008P^* lung tissue (40× original magnification; scale bar: 50 μm). WT/WT, *Rnf213^WT/WT^* mice; WT/KI, *Rnf213^WT/H4008P^* mice; KI/KI, *Rnf213^H4008P/H4008P^* mice.

To reveal the cause of death, we investigated E18.5 *Rnf213^H4008P/H4008P^* mouse fetuses. The hearts of E18.5 *Rnf213^H4008P/H4008P^* mice showed no obvious morphological and histological abnormalities compared with the hearts of *Rnf213^WT/WT^* and *Rnf213^WT/H4008P^* mice (Supplemental Figure 4, A and B). No abnormal vascular branching or hypoplasias were found in the aortic arch, thoracoabdominal aorta, and pulmonary artery (Supplemental Figure 4, C–E), and the arterial wall had no obvious disarrangement of elastic laminae in histology (Supplemental Figure 4F). In the tracheal cartilage, there was no obvious stenosis in structure (Supplemental Figure 4G). Histological analysis revealed that the lungs of *Rnf213^H4008P/H4008P^* mice had irregular surfaces and thicker alveolar walls, smaller airspaces, and more numerous small alveoli (Figure 4A). To elucidate the abnormal alveolar structure in *Rnf213^H4008P/H4008P^* mice, we investigated the expression of lung cell–specific hallmark RNAs (15) and the distribution of type I and type II alveolar epithelial cells, Clara cells, and lung fibroblasts. RNA-Seq–based transcriptome analysis of the left lung lobes of E18.5 *Rnf213^H4008P/H4008P^* mice revealed no significant differences in the expression of lung cell–specific hallmark RNAs (Supplemental Tables 1 and 2). However, the distribution of type I alveolar epithelial cells, type II alveolar epithelial cells, and Clara cells was disorganized in *Rnf213^H4008P/H4008P^* mice (Figure 4B).

### Transcriptome analysis reveals upregulation of immune and inflammatory responses and downregulation of cell proliferation in the lungs of E18.5 Rnf213^H4008P/H4008P^ mouse fetuses.

**Figure 5:**
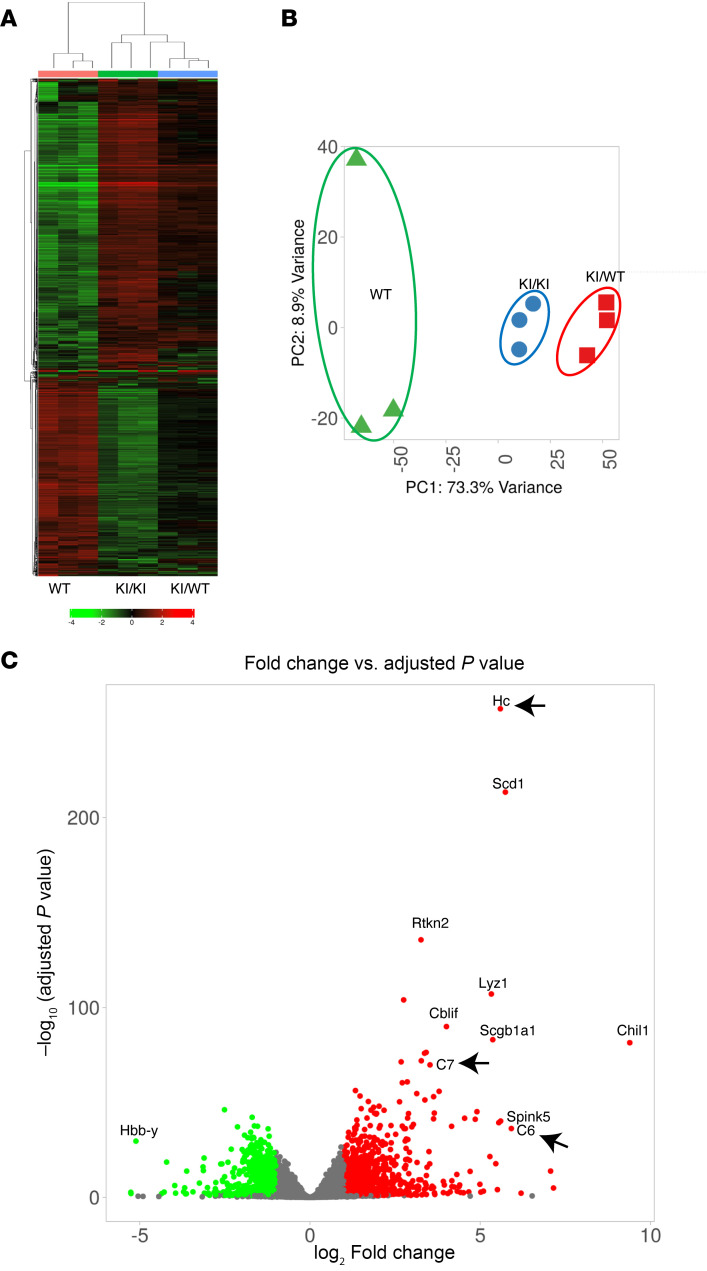
RNA-Seq analysis reveals different gene expression profiles between *Rnf213^WT/WT^* and *Rnf213^H4008P/H4008P^* mice. (**A**) Unsupervised hierarchical clustering using top 2,000 differentially expressed genes (DEGs) of transcriptomes from E18.5 *Rnf213^WT/WT^*, *Rnf213^WT/H4008P^*, and *Rnf213^H4008P/H4008P^* mouse lungs. (**B**) Principal component analysis same as in **A**. (**C**) DEGs shown as a volcano plot. Upregulated complement components (Hc, C6, and C7) are indicated by arrows. WT/WT, *Rnf213^WT/WT^* mice; WT/KI, *Rnf213^WT/H4008P^* mice; KI/KI, *Rnf213^H4008P/H4008P^* mice.

To study the etiology of the lethal structural abnormalities in the lungs of *Rnf213^H4008P/H4008P^* mice, we performed RNA-Seq–based transcriptome analysis of E18.5 lung tissues from the left lobe and compared differentially expressed genes (DEGs) between *Rnf213^WT/WT^*, *Rnf213^WT/H4008P^*, and *Rnf213^H4008P/H4008P^* mouse fetuses (Supplemental Table 3). The expression profiles of *Rnf213^WT/WT^*, *Rnf213^WT/H4008P^*, and *Rnf213^H4008P/H4008P^* mouse fetuses, analyzed by unsupervised hierarchical clustering and principal component analysis, clearly distinguished these genotypes (Figure 5, A and B). The expression profiles between *Rnf213^WT/H4008P^* and *Rnf213^H4008P/H4008P^* were relatively similar (Figure 5, A and B). A total of 1,842 upregulated genes and 1,544 downregulated genes were identified between *Rnf213^WT/WT^* and *Rnf213^H4008P/H4008P^* mice (Supplemental Figure 5). Furthermore, 3,007 upregulated genes and 2,753 downregulated genes were identified between *Rnf213^WT/H4008P^* and *Rnf213^H4008P/H4008P^* mice (Supplemental Figure 5). RNA-Seq transcriptome analysis revealed that complement components, such as hemolytic complement (*Hc*), *C6*, and *C7*, were significantly upregulated in *Rnf213^H4008P/H4008P^* mouse lung (Figure 5C). *Hc*, also known as *C5*, is a gene of the complement system and component of the innate immune system with important roles in inflammation, host homeostasis, and host defense against pathogens (16). C5 is cleaved into C5a and C5b. C5a acts as an anaphylatoxin, which promotes inflammation and recruits immune cells to the infection or injury site. C5b initiates the formation of the membrane attack complex by binding to C6 and C7. This complex inserts into the cell membrane and causes cell lysis (16, 17).

**Figure 6:**
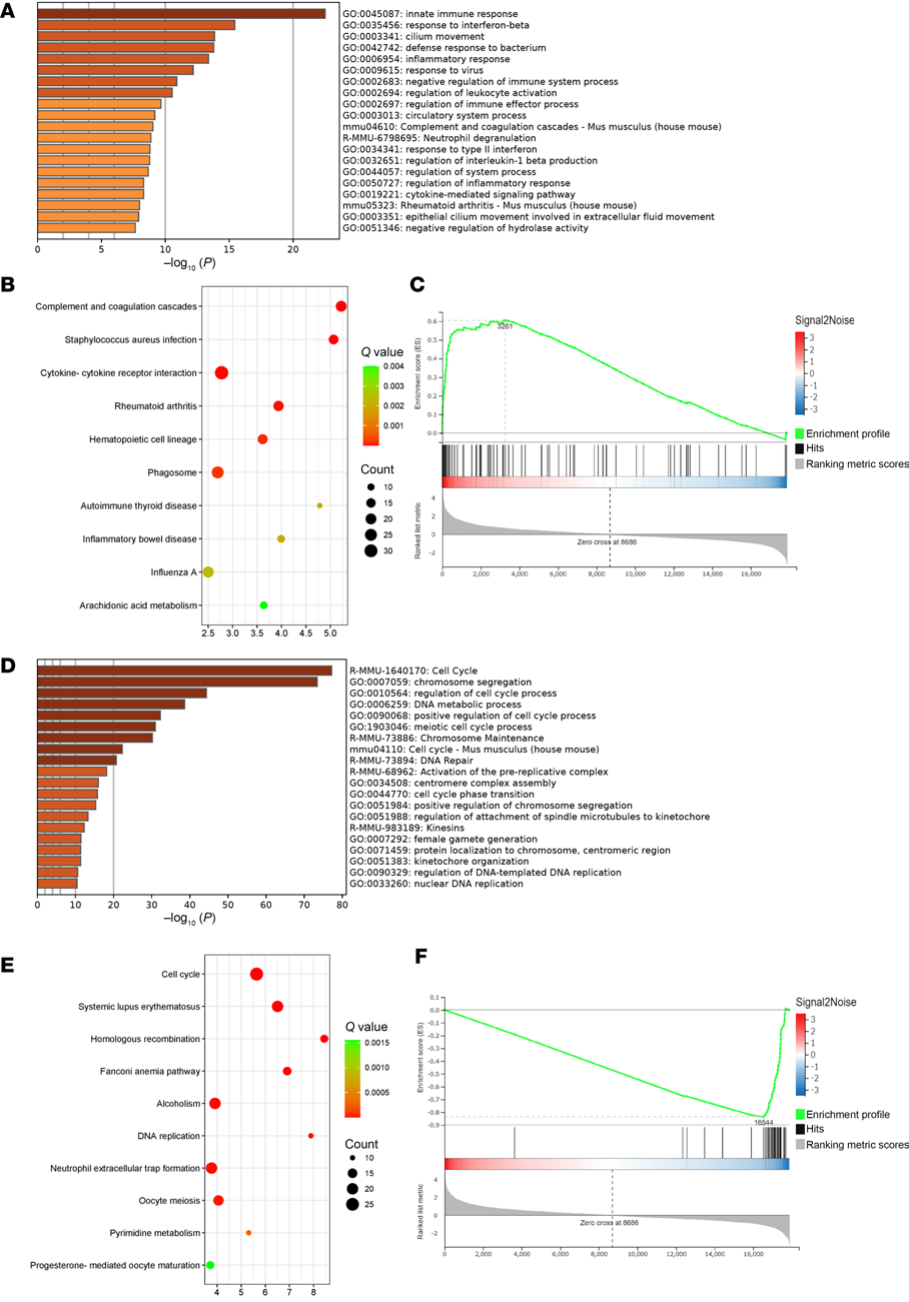
Annotation of DEGs and enrichment analysis. (**A**) Metascape analysis of DEGs (log_2_FC > 2 and FDR < 0.1) in the lungs of E18.5 *Rnf213^H4008P/H4008P^* mice compared with *Rnf213^WT/WT^* mice. Enriched annotations for upregulated genes. (**B**) Bubble chart shows KEGG pathway enrichment of upregulated DEGs. (**C**) GSEA profile of complement and coagulation cascade between E18.5 *Rnf213^H4008P/H4008P^* and *Rnf213^WT/WT^* mouse lungs. (**D**) Enriched annotations for downregulated genes with Metascape analysis. (**E**) Bubble chart shows KEGG pathway enrichment of downregulated DEGs. (**F**) GSEA enrichment profile of the cell cycle.

Enrichment analysis using Metascape (18), gene ontology (GO), and Kyoto Encyclopedia of Genes and Genomes (KEGG) revealed characteristic patterns. Genes involved in innate immunity were more highly expressed in *Rnf213^H4008P/H4008P^* than in *Rnf213^WT/WT^* mice (Figure 6A and Supplemental Table 4). KEGG enrichment analysis revealed enrichment of complement and coagulation cascades in *Rnf213^H4008P/H4008P^* mice (Figure 6B and Supplemental Table 5). GO enrichment analysis identified innate immune response in *Rnf213^H4008P/H4008P^* mice (Supplemental Figure 6A and Supplemental Table 6). Gene set enrichment analysis (GSEA) verified enrichment of genes involved in complement and coagulation cascades (Figure 6C and Supplemental Table 7). These results suggest activation of the innate immunity cascade, including the complement pathway, in *Rnf213^H4008P/H4008P^* lungs.

Metascape analysis of genes downregulated in *Rnf213^H4008P/H4008P^* compared with *Rnf213^WT/WT^* showed an enrichment of cell cycle–associated genes (Figure 6D and Supplemental Table 8). Consistently, KEGG and GO enrichment analysis and GSEA showed enrichment of cell cycle–related genes in *Rnf213^H4008P/H4008P^* (Figure 6, E and F; Supplemental Figure 6B; and Supplemental Tables 5–8). These results suggest defective cell proliferation, development, and maturation in the *Rnf213^H4008P/H4008P^* lung.

Although lung morphology was unaltered in heterozygous *Rnf213^WT/H4008P^* mice, gene expression pattern was similar to that of *Rnf213^H4008P/H4008P^*. Activation of the innate immunity cascade, including the complement and coagulation cascade, and downregulation of cell proliferation were observed (Supplemental Figure 7, A–F, and Supplemental Tables 9 and 10).

**Figure 7:**
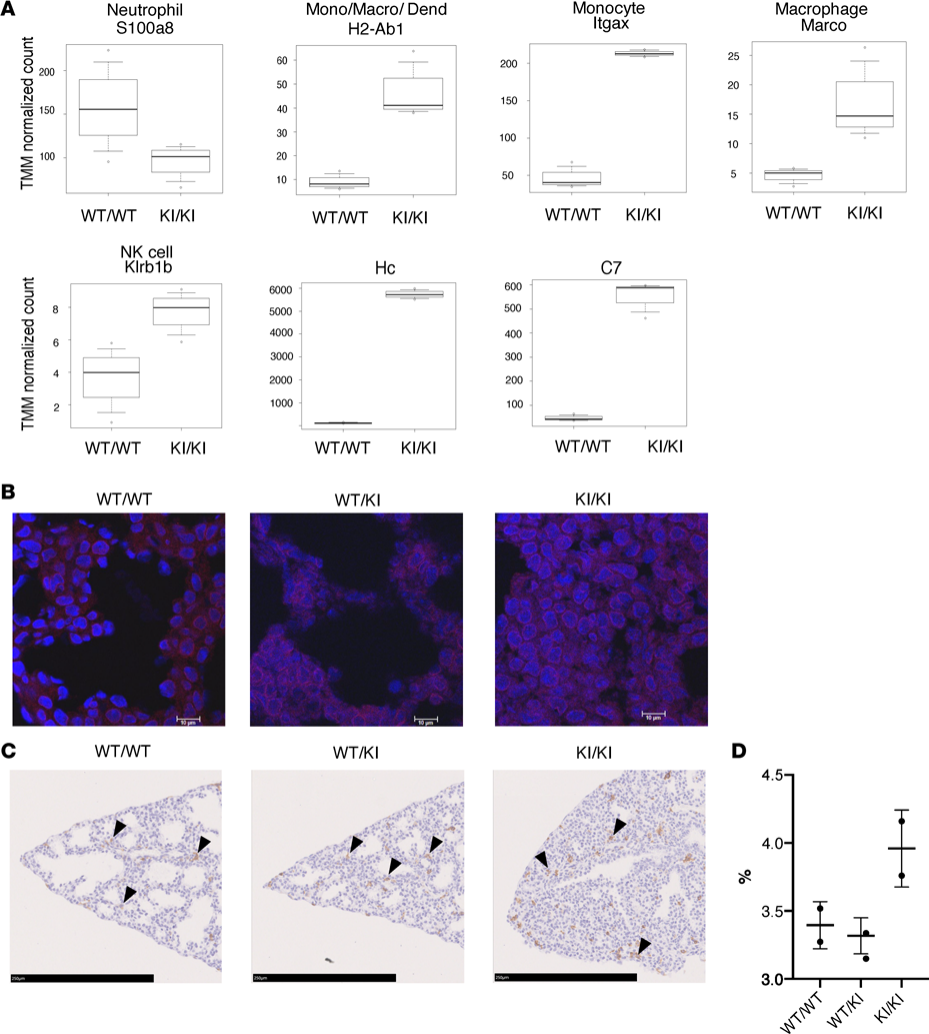
Expression of genes involved in innate immunity and complement system in the E18.5 lung. (**A**) Expression of genes involved in innate immunity and complement system in the lungs of E18.5 *Rnf213^H4008P/H4008P^* mice compared with *Rnf213^WT/WT^* mice. In the upper and lower row, the representative marker of white blood cell subpopulation and protein name are listed, respectively. Mono/Macro/Dend, monocyte/macrophage/dendritic cell; TMM, EdgeR’s trimmed mean of M values. (**B**) Immunohistochemistry staining for C5a (red) and cell nuclei (blue, DAPI) (63× original magnification, scale bar: 10 μm). (**C**) Arrows, F4/80-positive cells (M1 and M2 macrophages) were stained dark brown, whereas the nuclei were stained purple in E18.5 *Rnf213^WT/WT^*, *Rnf213^WT/H4008P^*, and *Rnf213^H4008P/H4008P^* lungs (20× original magnification, scale bar: 250 μm). (**D**) F4/80-positive cells (M1 and M2 macrophages) calculated as percentage of total area per caudal lobe tissue, with comparison between genotypes. (WT/WT, *n* = 2; WT/KI, *n* = 2; and KI/KI, *n* = 2.) Box plots show the interquartile range, median (line), and minimum and maximum (whiskers). Only 2 samples were available; therefore, statistical analysis was not performed. WT/WT, *Rnf213^WT/WT^* mice; WT/KI, *Rnf213^WT/H4008P^* mice; KI/KI, *Rnf213^H4008P/H4008P^* mice.

Among the genes associated with innate immune response (GO:0045087) (Supplemental Table 11) and genes characterizing immune cells (Supplemental Table 12) in the lung, the expressions of representative genes that characterize the cells involved in innate immunity are shown as a box blot (Figure 7A). S100A8 is a calcium-binding protein that is highly expressed in neutrophils and plays an important role in neutrophil-mediated inflammation (19). H2-Ab1 is a mouse MHC class II molecule that is mainly expressed in antigen-presenting cells, such as monocytes, dendritic cells, and macrophages. It contributes to the immune response by presenting antigens to CD4^+^ T cells (20). *ITGAX*, also known as *CD11c*, encodes a protein involved in immune cell adhesion and phagocytosis and is expressed on monocytes, macrophages, and dendritic cells in mice (21). The scavenger receptor MARCO plays a crucial role in alveolar macrophage function and innate immunity in mice (22). *Klrb1b*, also known as *CD161*, encodes the NK-1.1 surface antigen expressed on NK cells (23). Among the cells involved in innate immunity, monocyte-, macrophage-, or NK cell–associated genes were highly expressed in the *Rnf213^H4008P/H4008P^* lung. In addition to data obtained from RNA-Seq analysis, immunohistological analysis revealed increased expression of C5a in *Rnf213^H4008P/H4008P^* and *Rnf213^WT/H4008P^* lungs compared with that in *Rnf213^WT/WT^* lung (Figure 7B). Complement plays an important role in regulating macrophage function and migration during inflammation (24). Alveolar macrophage–derived serine proteases cleave epithelial cell–produced C5 to C5a, which initiates inflammatory signaling when bound to its receptor C5aR (25). We analyzed F4/80-expressing macrophages to examine the distribution of macrophages in the embryonic mouse lung (26). The number of F4/80-positive macrophages was higher in the lung interstitium of *Rnf213^H4008P/H4008P^* mice than in the lungs of *Rnf213^WT/WT^* and *Rnf213^WT/H4008P^* mice (Figure 7, C and D). The numbers of T cells and apoptotic cells analyzed by CD3 antibody and TUNEL assay, respectively, did not increase in all genotypes (Supplemental Figure 8, A and B).

## Discussion

In this study, we identified 2 potentially novel disease-associated variants in *RNF213* in patients with MAS and MMD from 2 independent families. Genome-wide association studies have revealed the association of single nucleotide polymorphisms at the *RNF213* locus in patients with MMD from East Asia (2, 27–29). *RNF213* p.R4810K is a founder variant of MMD that is common in Japan (1.6% in ToMMo 54KJPN) and Asia, with low-penetrance, autosomal dominant form of inheritance, and 74%–90% of the patients in Japan carry this variant (2, 27). The *RNF213* variant is a risk factor for various vascular diseases, including vascular occlusive disease (4) and atherosclerosis (28). In a report, 17% of 63 young adult patients with MMD had asymptomatic arterial narrowing (>50%) at multiple sites, including the coronary, superior mesenteric, celiac, renal, and internal iliac arteries (11). Notably, in homozygous *RNF213* p.R4810K, 3/4 patients had extracerebral arteriopathy (11). Recently, rare *RNF213* variants, located in the RING domain and a discrete region distal to the RING domain, were detected by WES in childhood-onset MMD with diffuse occlusive vasculopathy, including the abdominal aorta and renal, iliac, and femoral arteries (29).

*Rnf213^WT/H4008P^* mice did not exhibit abnormal vascular structure; however, *Rnf213^H4008P/H4008P^* mice died from lung dysfunction. *Rnf213* p.R4810K heterozygous KI mice grew normally, and even *Rnf213* p.R4810K homozygous KI mice had no significant differences in brain artery structure and the structure of the circle of Willis (30). However, *Rnf213* p.R4810K overexpression attenuated angiogenesis in vitro (31). Therefore, *Rnf213* p.R4810K was considered a variant that exerts function with environmental factors, such as infection, autoimmunity, and inflammation, contributing to disease development (31). *Rnf213*-knockout mice exhibited no abnormalities in cervical or intracranial arteries or the circle of Willis; however, after internal carotid artery ligation–induced ischemia, the intimal and medial layers of the vessels were significantly thinned (32). In this study, no vascular phenotype was observed under normal conditions. Further studies are needed to determine whether *Rnf213^WT/H4008P^* mice develop vascular lesions after ischemia, inflammation, hypoxia, and infection. A noteworthy phenotype observed in *Rnf213^WT/H4008P^* mice was a lack of care for pups. Research focusing on the psychological effects of *Rnf213^WT/H4008P^* variants is of interest, particularly in relation to molecular changes such as increased innate immunity and inflammation, as revealed by transcriptome analysis.

We revealed that *Rnf213* p.H4008P regulates the expression of molecules involved in inflammation, immune response, complement system, and cell proliferation. Transcriptome analyses of samples from MMD middle cerebral artery showed increased expression of genes involved in the immune response, antigen processing and presentation, dendritic cell pathway, and cytokine pathway including interleukin-12 pathway, as well as decreased expression of genes related to DNA repair and oxidative phosphorylation (33). This finding is consistent with those observed in the transcriptome analysis of lung tissues from *Rnf213^H4008P/H4008P^* mice. However, because our results were obtained from different tissues, further analyses using blood vessels are required for comprehensive evaluation. Regarding innate immunity, a study indicated that *RNF213* plays a key role in familial chronic urticaria with hypercytokinemia (34), and the *RNF213* RING domain variants increased NF-κB activity and induced apoptosis with caspase-3 activation in HEK293T cells (35). NF-κB is one of the key signaling molecules for monocyte and macrophage function (36). The lungs of *Rnf213^H4008P/H4008P^* mice showed an increased number of macrophages. We hypothesize that the abnormal lung morphology in *Rnf213^H4008P/H4008P^* mice is caused by activation of the inflammatory cascade and *Rnf213^H4008P/H4008P^* is related to the immune response pathway and the enhancement of inflammation mediated by the NF-κB pathway.

Our transcriptome analysis revealed that *Rnf213^H4008P/H4008P^* increased stearoyl-CoA desaturase 1 (*Scd1*) expression, suggesting an effect on lipid metabolism. *RNF213* protected cells from saturated fatty acid–induced lipotoxicity by inhibiting Scd1 activity and affecting the cellular ability to store lipids in lipid droplets (37). Based on our findings and previous research, we hypothesize that lipotoxicity induces inflammatory pathways and affects tissue development. Compared with *Rnf213^WT/WT^*, the neutrophil count in *Rnf213^H4008P/H4008P^* decreased on RNA-Seq, as indicated by a decrease in *S100a8* expression. However, the monocyte and macrophage count increased, as indicated by the increased expression of *H2-Ab1*, *Itgax*, and *Marco*. Furthermore, the number of F4/80-positive macrophages increased in *Rnf213^H4008P/H4008P^*. S100a9 in neutrophils acts on prostaglandin receptors in macrophages, thereby conferring M2 macrophage polarization. F4/80 is a marker that is primarily expressed on the surface of M1 (inflammatory) and M2 (antiinflammatory) macrophages in mature mice (38). Therefore, we speculated that a decrease in neutrophil count may activate M1 macrophage polarization. Furthermore, lung inflammation may occur secondary to lung damage of other etiologies. We believe that lung inflammation is a primary event because the pups died of lung failure immediately after birth. If lung inflammation occurred as a secondary phenomenon, it may have resulted from changes in lung circulation during embryogenesis. However, we think this is unlikely because the structure of the heart and major arteries remained normal.

This is the first report to our knowledge on an *Rnf213* RING domain missense variant that promotes innate immunity and the complement cascade and alters lipid metabolism in lung tissues without any vascular phenotype. This suggests the complexity of tissue-dependent *RNF213* function or species-dependent tissue susceptibility. Interestingly, the abnormal activation of innate immunity also occurred in tissues other than the lungs. However, we did not investigate the possible activation of innate immunity throughout the body because the phenotypes were observed only in the lungs of these mice. We therefore recommend future studies to investigate the vasculature from this point of view. Environmental factors may contribute to the development of MMD with the *RNF213* R4810K variant. Evaluation of *Rnf213^H4008P/H4008P^* mice under stress, such as ischemia or inflammation, or during the early embryonic stages may provide insights into the function of *RNF213* in the vascular system. Further studies are necessary to elucidate the mechanism of these signaling pathways in mediating the effects of heterozygous *RNF213* pathogenic variants.

## Methods

### Sex as a biological variable.

This study included 2 male patients. Given the rarity of the disease, findings are presented for only 1 sex; however, there are no sex differences in the epidemiology of the disease. The mouse experiments included both male and female mice. Genetic variants were studied equally, and sex was not considered a biological variable in terms of the effect of genetic variants on the function of *RNF213*.

### WES analysis.

Genomic DNA was extracted from the peripheral blood using QIAamp DNA Mini Kit (QIAGEN), and WES analysis was performed using SureSelect Human All Exon V6 (Agilent Technologies) for capture and HiSeq 2500 (Illumina) for sequencing with 101 bp paired-end reads. Reads were aligned to GRC37 using Burrows-Wheeler Aligner (https://bio-bwa.sourceforge.net/). Variants were called using GATK Unified Genotyper and ANNOVAR (https://annovar.openbioinformatics.org/en/latest/). Variants were filtered with minor allele frequency greater than 0.01 using the 1,000 Genomes Project database (http://www.1000genomes.org/); NIH National Heart, Lung, and Blood Institute GO Exome Sequencing Project; ExAC; NIH dbSNP (https://www.ncbi.nlm.nih.gov/snp/); Human Genetic Variation Database (https://www.hgvd.genome.med.kyoto-u.ac.jp); and a comprehensive Japanese genetic variation database (TogoVar, https://grch38.togovar.org/). The detected *RNF213* variants were validated by Sanger sequencing. For each detected *RNF213* variant, prediction of pathogenicity was assessed using Sorting Intolerant from Tolerant (https://sift.bii.a-star.edu.sg), CADD (http://cadd.gs.washington.edu), and MutationTaster (http://www.mutationtaster.org).

### Generation of Rnf213 p. His4008Pro (H4008P) variant KI mice

. *Rnf213*-KI mice were generated using the cloning-free CRISPR/Cas9 genome-editing system, as described (39). Both crRNA (CRISPR RNA; 5′-gaacugggcgugcuucucaaGUUUUAGAGCUAUGCUGUUUUG-3′, where the lowercase letters indicate the *Rnf213* target sequence) and tracrRNA (trans-activating CRISPR RNA; 5′-AAACAGCAUAGCAAGUUAAAAUAAGGCUAGUCCGUUAUCAACUUGAAAAAGUGGCACCGAGUCGGUGCU-3′) were designed as previously described (33).

Recombinant Cas9 protein (Z2641N, Takara Bio), crRNA, tracrRNA, and the repair template ssODN (5′-AAGTATTGGGCAACACATTCTACAATTCTTCTGTCACGAGTTCTCCCTCCCTCCTAAGGAAAGCCATTGAGAAGCCCGCCCAGTTCCGGCACATGTGCAACAGCTTTTTTGTGGACCTTGTTTCTACCATGTGCTTCAAGGATAACACGC-3′) were coinjected into the pronuclei of C57BL6/J fertilized eggs. Founder offspring that harbored the mutant allele were selected and crossed to wild-type C57BL6/J mice (CLEA Japan) to establish the *Rnf213^H4008P^* mouse line.

### MR neuroimaging.

In vivo imaging was performed using 7 T MRI by Live Animal Imaging Center at Central Institute for Experimental Animals. MRI was performed under isoflurane anesthesia, according to the guidelines and policies for animal surgery provided by the animal study committees of Central Institute for Experimental Animals.

### Ink injection.

Pregnant females were euthanized by CO_2_ inhalation, and the embryos were quickly dissected with yolk sac and placenta attached. E18.5 embryos were placed into a Petri dish with warm PBS/heparin (20 mg/mL) (3 drops in 3 mL PBS). Under a dissecting scope (LW-820, WRAYMER), 0.1 mL of India ink (Kiwaguro, Sailor) was injected to fill the intravascular compartment through the umbilical artery of placenta using a 30-gauge nanoinjection needle (Saito Medical Instruments Inc.) (40). Then, the embryos were dissected from surrounding membranes and immersion-fixed in 4% paraformaldehyde/PBS overnight.

### Benzyl alcohol/benzyl benzoate tissue clearing.

Immersion-fixed embryos were washed with PBS and stored in 70% ethanol/PBS. The embryos were dissected out to examine the structure of the aortic arch, major artery, and pulmonary vessels. The organs were dehydrated and cleared in benzyl alcohol/benzyl benzoate solution (1 volume benzyl benzoate: 1 volume benzyl alcohol) (41, 42). Images were taken under a stereoscopic microscope (LW-820, WRAYMER).

### Aortic ring assay.

For the aortic ring assay, 8-week-old mice were euthanized and bled out, and their thoracic aortas were removed and cut into round, 1 mm slices in cold sterile PBS. Aortas were embedded in collagen gel culture (Cellmatrix type-IA, Nitta gelatin) with a medium (EGM-2 Bullet Kit, VEGF 50 ng/mL) (Lonza) in 48-well plates (14). The VEGF-induced microvascular sprouting from the cut surfaces were observed on day 14 using a microscope (Nikon SMZ1000)

### Immunohistochemistry.

The aortic arteries, hearts, and lungs of E18.5 embryos dissected from pregnant mice were fixed in 4% paraformaldehyde/PBS at 4°C overnight, sequentially dehydrated in 30% sucrose in PBS, and frozen in Tissue Tek OCT (Sakura). The formalin-fixed, paraffin-embedded tissue sections were deparaffinized with xylene and dehydrated through graded alcohol. For antigen retrieval, the sections were processed in 0.01 M citric acid (pH 6.0) for 15 minutes in a microwave oven, washed with PBS, and blocked with 10% goat serum in 0.2% Triton X-100/PBS at room temperature for 1 hour. Immunohistochemical staining was performed using monoclonal primary antibodies diluted with 0.2% Triton X-100/PBS against proSP-C (AB3786, 1:1,000, Sigma-Aldrich), CC10 (sc-365992, 1:100, Santa Cruz Biotechnology), podoplanin (sc-53533, 1:100, Santa Cruz Biotechnology), SMA (sc-53142, 1:600, Santa Cruz Biotechnology), and C5a (bs-0324-TR, 1:50, Bioss). The slides were washed with PBS, then incubated with secondary antibody for 1 hour at room temperature: Alexa Fluor 594 goat anti-rabbit IgG (A-11037, 1:200, Invitrogen), Alexa Fluor 568 goat anti-mouse IgG (A-11004, 1:200, Invitrogen), and Alexa Fluor 568 goat anti-hamster IgG (A-21112, 1:200, Invitrogen). After incubation, the slides were mounted using VECTASHIELD Antifade Mounting Medium with DAPI (Vector Laboratories). The images were acquired with a fluorescence microscope equipped with phase-contrast setting (BZ-X700, Keyence). For conventional histology, paraffin sections were stained with hematoxylin and eosin (HE), periodic acid–Schiff, and Elastica van Gieson. TUNEL assay and immunohistochemical staining with CD3 (ab134096, Abcam) and F4/80 antibodies (CL8940AP, Cedarlane Laboratories) were performed by Morphotechnology Co., Ltd. F4/80 antibody stained M1 and M2 macrophages in dark brown, and HE staining stained the nuclei purple. The percentage of F4/80 antibody–stained macrophage (M1 and M2) area was calculated as the ratio of staining area to whole caudal right lobe area.

### Staining of trachea cartilage.

Tracheas were dissected from E18.5 embryos, fixed in 4% paraformaldehyde/PBS, and stored in 70% ethanol/PBS as described in the earlier section. Tracheas were digested with 2% KOH, stained with alcian blue (pH 2.5) (Muto Pure Chemical Co.) (43), and observed under a stereoscopic microscope (LW-820, WRAYMER).

### RNA-Seq.

E18.5 embryos were removed from the uteri of CO_2_-anesthetized pregnant mice, and the left lungs were removed and flash-frozen. To avoid secondary lung inflammation during the procedure, we used littermate pups, confirmed the presence of a heartbeat, and quickly removed the lungs for investigation. Total RNA was qualified and quantified using Agilent Technologies RNA 6000 Nano Kit and Agilent Technologies 2100 bioanalyzer. Library preparation and RNA-Seq were performed using the DNBSEQ Sequencing platform (BGI Genomics) at paired-end 100 bp read length.

### RNA-Seq data analysis.

Quality control was performed on the raw reads by BGI using the filtering software SOAPnuke v1.5.2. Clean reads were mapped to the reference genome (*Mus musculus*, NCBI, GCF_000001635.27_GRCm39) by HISAT2 v2.0.4/BOWTIE2v. Read counts were normalized to transcripts per kilobase of exon model per million mapped reads. The *q* value was obtained by false discovery rate correction of the *P* value. DEGs (*q* ≤ 0.1, log_2_FC ≥ 2) were analyzed by DEseq2 software. GSEA, heatmap, volcano plot, GO, principal component analysis, and KEGG analyses were performed using Dr. Tom multiomics data mining system (BGI Genomics). iDEP was used to perform hierarchical clustering with a heatmap and principal component analysis and construct volcano plots (44). Enrichment analysis using DEGs (*q* ≤ 0.1, log_2_FC ≥ 2) was performed using Metascape (18). GSEA was performed using GSEA v4.1.0 (45).

### Statistics.

All statistical analyses were conducted using EasyR or Prism 7 software (GraphPad Software). Data are shown as means ± SEM and statistically analyzed using the Mann-Whitney *U* test. One-way ANOVA was used to compare 3 groups. For all analyses, *P* < 0.05 was considered statistically significant.

### Study approval.

All animal experiments were performed with the approval of the Institutional Animal Care and Use Committee of Institute of Science Tokyo (A2021-006A) and followed the Animal Research: Reporting of In Vivo Experiments guidelines. The Ethics Committee of Institute of Science Tokyo approved this study (G2000-103). Medical reports, blood samples for genetic testing, and imaging data were obtained after written informed consent was provided by the patients or their legal guardians, in accordance with the principles of the Declaration of Helsinki. Genetic testing was approved by the National Research Institute for Child Health and Development, Initiative on Rare and Undiagnosed Disease in Pediatrics, Japan.

### Data availability.

Data are available in the Supporting Data Values file and are available upon request. RNA-Seq data can be accessed at the NCBI’s Gene Expression Omnibus database (GSE282828).

## Author contributions

AK and MT conceived the study and developed the methodology. AK, KU, KK, and YH conducted the investigation. T Mizuno, ET, SH, and TU provided essential resources. T Morio provided scientific supervision. AK performed data visualization. MT supervised the overall study. AK and MT were responsible for project administration, and both contributed to reviewing and editing the manuscript.

## Supplementary Material

Supplemental data

Supplemental tables 1-12

Supporting data values

## Version 1 06/09/2025

Electronic publication
