# Supplementary material for: De novo variant in RING finger protein 213 causes systemic vasculopathy
Source: JCI Insight. 2025 Jun 9;10(11):e190094. doi: 10.1172/jci.insight.190094 (PMC12220945; doi:10.1172/jci.insight.190094)
Supplement: Supplemental data [file jciinsight-10-190094-s072.pdf]

Supplementary Figure 1

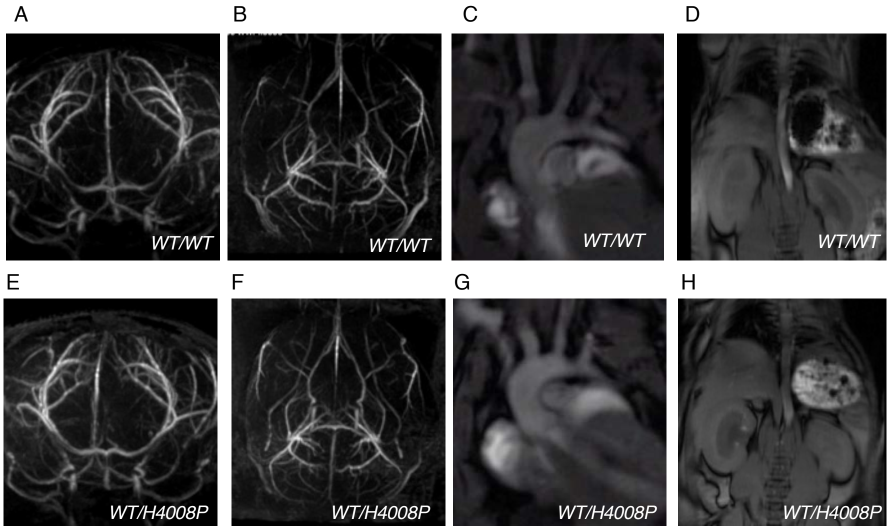

Supplementary Figure 1. MR angiography
MR angiography of 6 months old *Rnf213*$^{WT/H4008P}$ mice (E-H) compared to the *Rnf213*$^{WT/WT}$(A-D). In the frontal (A)(E) and axial view (B)(F) of cerebral arteries and aortic arch (C)(G) to descending aorta(D)(H). No obvious abnormalities were detected between *Rnf213*$^{WT/H4008P}$ and *Rnf213*$^{WT/WT}$ mice.

Supplementary Figure 2

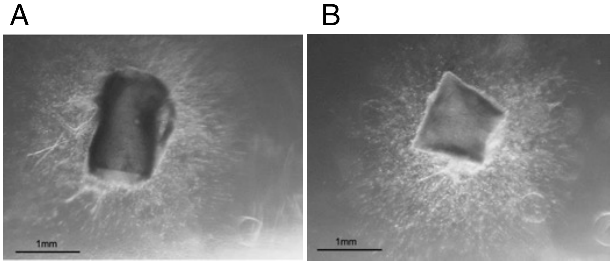

Supplementary Figure 2. Aortic ring assay
Aorta from 8 weeks old mice littermate ((A) *Rnf213*$^{WT/WT}$, (B) *Rnf213* $^{WT/H4008P}$) were cut into round 1mm slices and perform organ culture in Matrigel with a medium (EGM-2 Bullet Kit, VEGF 50ng/mL) (Lonza). The formation of capillaries from the cut surfaces were observed at day 14. Scale bars: 1mm.

Supplementary Figure 3

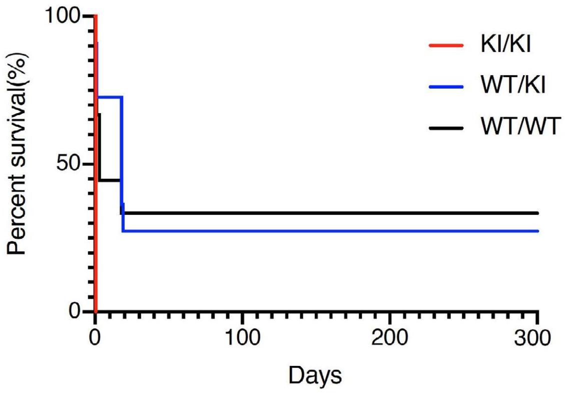

Supplementary Figure 3. Survival of *Rnf213*$^{WT/H4008P}$ littermate.
The pups were fostered by own biological *Rnf213* $^{WT/H4008P}$ mother.
Kaplan-Meier survival curves. (WT/WT, n = 9; WT/KI, n = 11; KI/KI, n = 8).
Abbreviations; WT/WT, *Rnf213* $^{WT/WT}$ mice; WT/KI *Rnf213* $^{WT/H4008P}$ mice; KI/KI, *Rnf213*$^{H4008P/H4008P}$ mice

Supplementary Figure 4

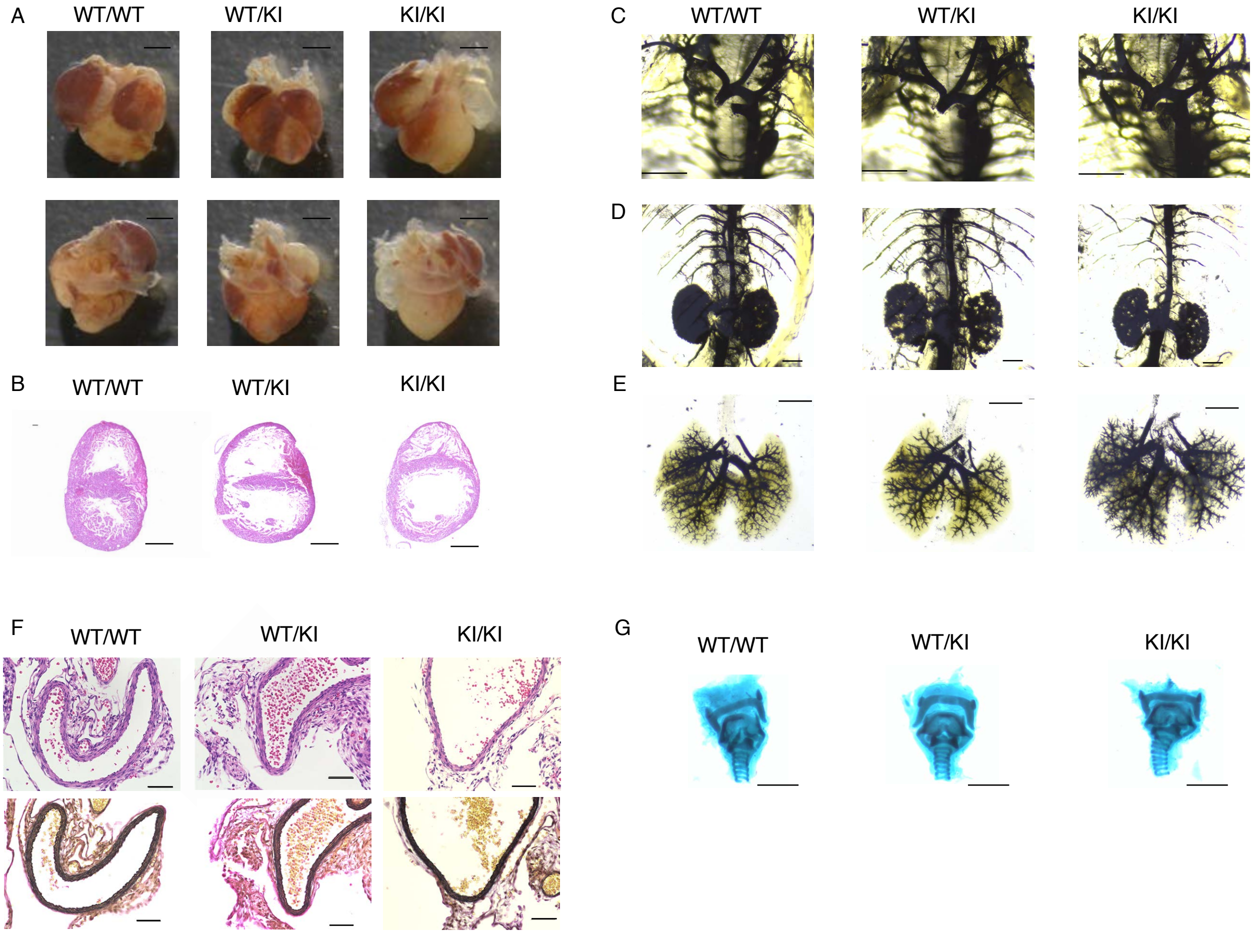

Supplementary Figure 4. Phenotype of E18.5 *Rnf213* $^{WT/WT,}$ *Rnf213* $^{WT/H4008P}$ and *Rnf213* $^{H4008P/H4008P}$ mice

(A) Phenotypic comparison of the heart of E18.5*Rnf213* $^{WT/WT}$, *Rnf213* $^{WT/H4008P}$ and *Rnf213* $^{H4008P/H4008P}$ mice. Scale bar, 1mm. (B) H&E staining of the heart at E18.5. Scale bar, 1mm. (C) Comparison of Ink injected Ink injected aortic arch and (D) thoracic aorta and (E) abdominal aorta pulmonary arteries Scale bar, 1mm. (F) HE/EVG staining image of thoratic artery in E18.5 *Rnf213* $^{WT/WT}$, *Rnf213* $^{WT/H4008P}$ and *Rnf213* $^{H4008P/H4008P}$ mice, Scale bar, 50µm. (G) Alcian blue staining of tracheal cartilage in E18.5 *Rnf213* $^{WT/WT}$, *Rnf213* $^{WT/H4008P}$ and *Rnf213* $^{H4008P/H4008P}$ mice, scale bar, 1mm.

Abbreviations; WT/WT, *Rnf213* $^{WT/WT}$mice; WT/KI, *Rnf213* $^{WT/H4008P}$ mice; KI/KI, *Rnf213*$^{H4008P/H4008P}$

Supplementary Figure 5

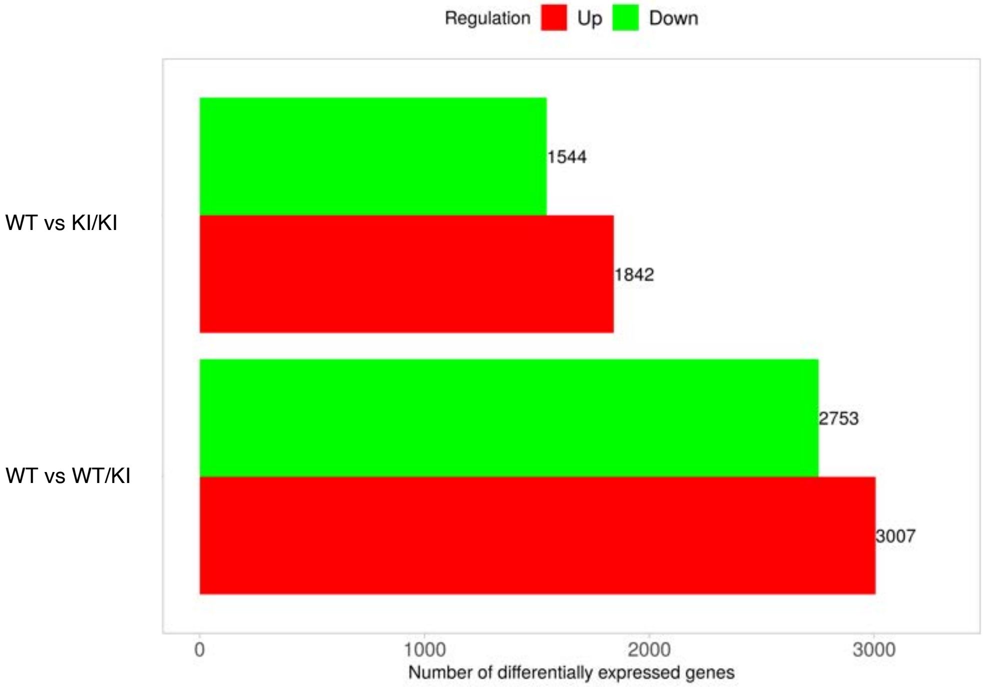

Supplementary Figure 5. Number of differentially expressing genes (DEG)
Number of DEGs between *Rnf213*$^{WT/WT}$(WT), *Rnf213*$^{WT/H4008P}$ (WT/KI), *Rnf213* $^{H4008P/H4008P}$(KI/KI), LogFC>2, FDR<0.1.
Abbreviations; WT, *Rnf213* $^{WT/WT}$mice; WT/KI, *Rnf213* $^{WT/H4008P}$ mice; KI/KI, *Rnf213*$^{H4008P/H4008P}$mice.

Supplementary Figure 6

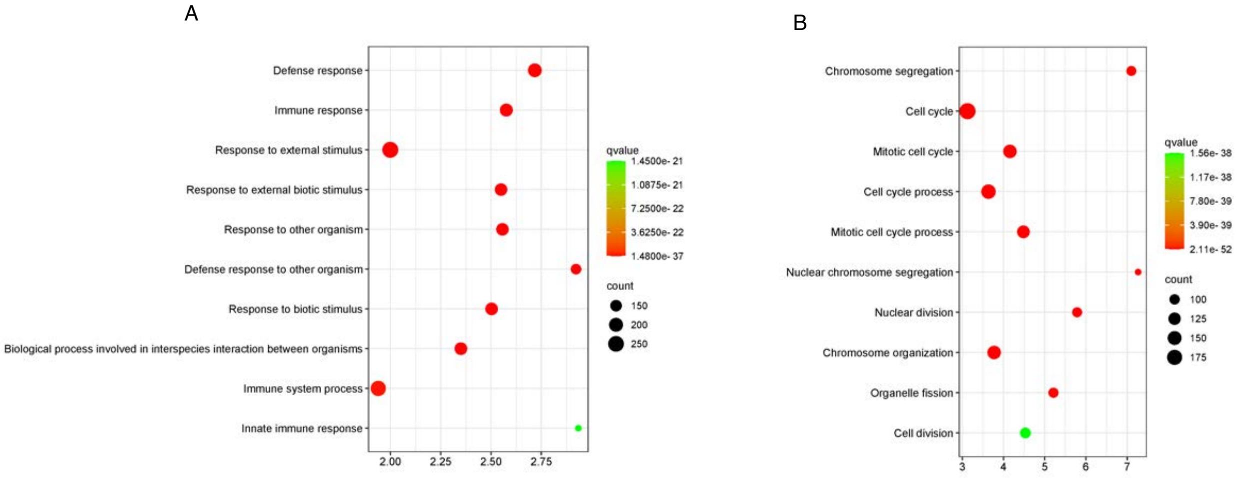

Supplementary Figure 6. Annotation of differentially expressing genes and enrichment analysis

(A) Bubble chart of enriched annotations for GO upregulated DEGs and (B) downregulated DEGs in the lung from E18.5 *Rnf213* $^{H4008P/H4008P}$ compared to *Rnf213* $^{WT/WT}$ mice.

Abbreviations; WT/WT, *Rnf213* $^{WT/WT}$ mice; WT/KI, *Rnf213* $^{WT/H4008P}$ mice; KI/KI, *Rnf213* $^{H4008P/H4008P}$ mice

Supplementary Figure 7

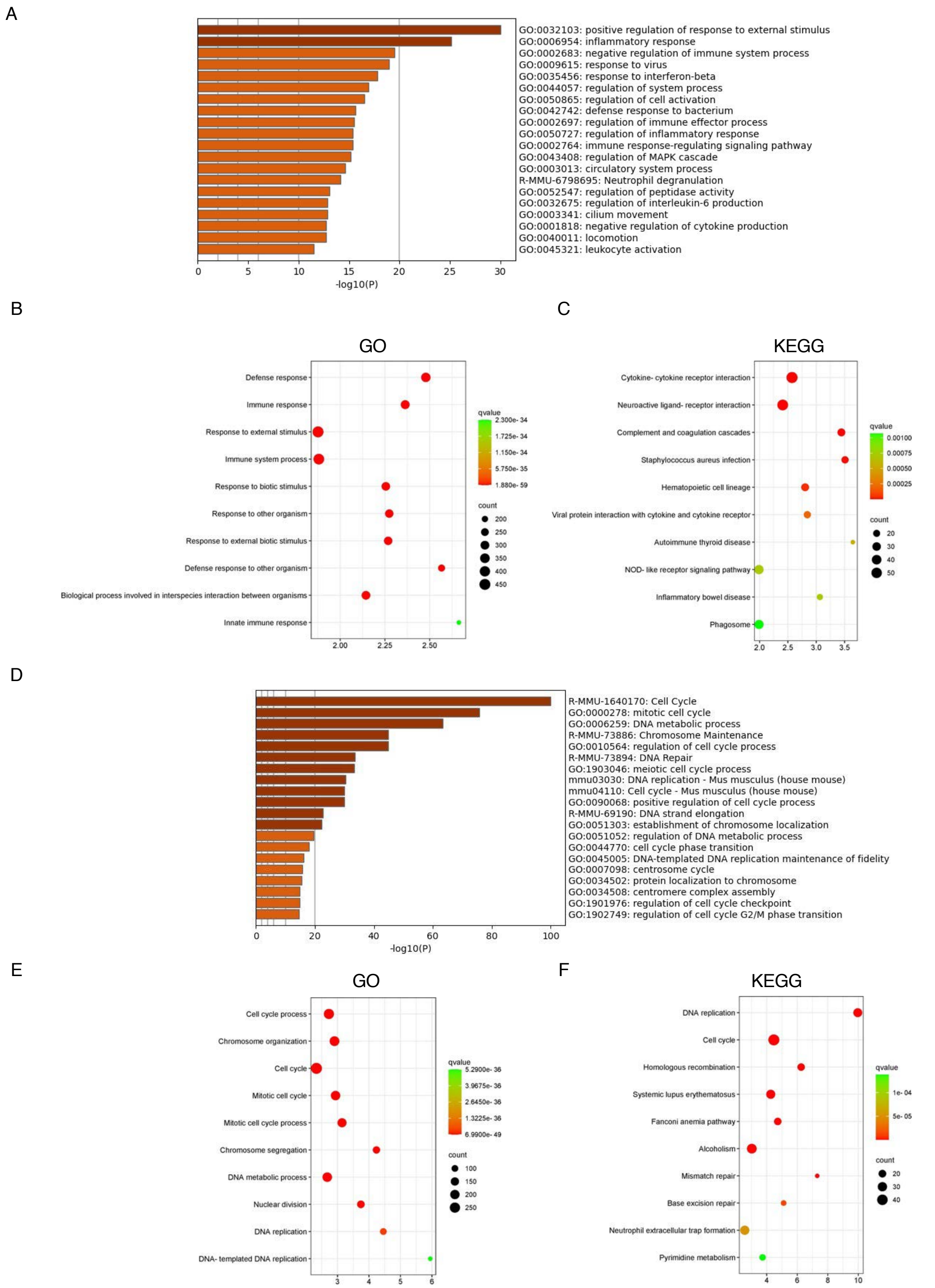

Supplementary Figure 7. Annotation of differentially expressing gens and enrichment analysis

(A) Metascape analysis using DEGs (LogFC >2 and FDR<0.1) in the lung from E18.5*Rnf213*$^{WT/H4008P}$ *compared to Rnf213*$^{WT/WT}$. Enriched annotations for upregulated genes. (B) Bubble chart for GO of upregulated DEGs. (C) Bubble chart for KEGG pathway of upregulated DEGs. (D) Enriched annotations for down regulated genes by Metascape analysis. (E) Bubble chart for GO of down regulated DEGs (E) Bubble chart for KEGG pathway of down regulated DEGs. Abbreviations; WT/WT, *Rnf213*$^{WT/WT}$ mice; WT/KI, *Rnf213*$^{WT/H4008P}$ mice; KI/KI, *Rnf213*$^{H4008P/H4008P}$

Supplementary Figure 8

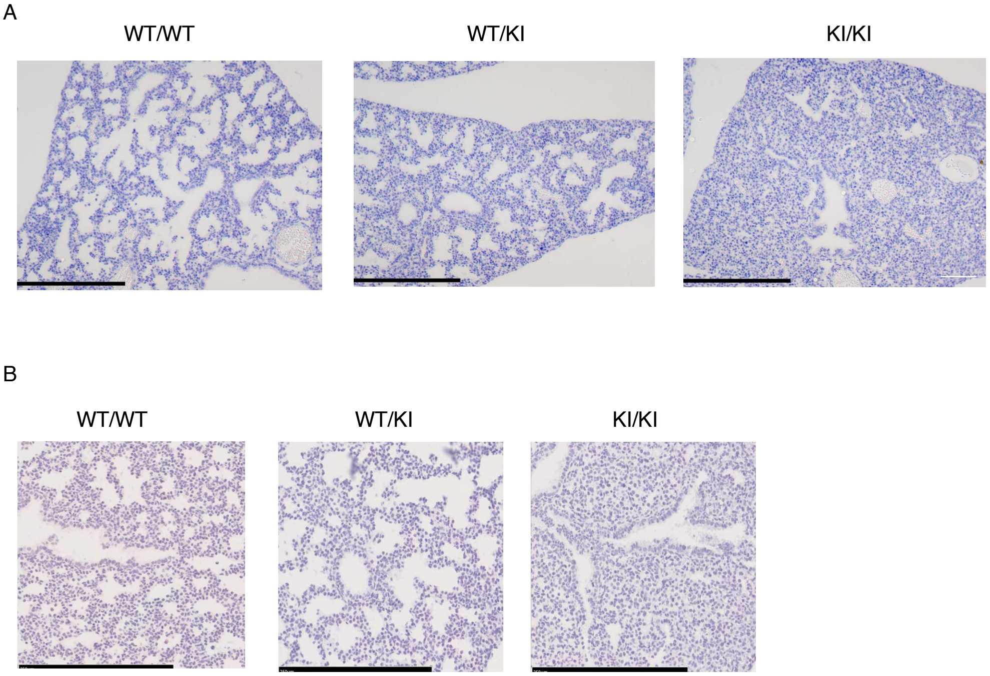

Supplementary Figure 8 . Immunohistochemical staining and TUNEL assay
(A) Immunohistochemical staining for CD3 staining, scale bar 250µm. (B) TUNEL staining, scale bar, 250µm. Abbreviations; WT/WT, *Rnf213* $^{WT/WT}$mice; WT/KI, *Rnf213* $^{WT/H4008P}$ mice; KI/KI, *Rnf213* $^{H4008P/H4008P}$mice
